# Epidemiology of basal and cutaneous squamous cell carcinoma in the U.K. 2013–15: a cohort study

**DOI:** 10.1111/bjd.17873

**Published:** 2019-05-06

**Authors:** Z.C. Venables, T. Nijsten, K.F. Wong, P. Autier, J. Broggio, A. Deas, C.A. Harwood, L.M. Hollestein, S.M. Langan, E. Morgan, C.M. Proby, J. Rashbass, I.M. Leigh

**Affiliations:** ^1^ Department of Dermatology Leicester Royal Infirmary Leicester U.K.; ^2^ Public Health England London Region London U.K.; ^3^ Barts and The London School of Medicine and Dentistry London U.K.; ^4^ Department of Dermatology Erasmus Medical Center Burg Jacobsplein 51, Rotterdam 3015CA the Netherlands; ^5^ International Prevention Research Institute Lyon France; ^6^ Information Services Division NHS National Services Scotland Glasgow U.K.; ^7^ Blizard Institute, Barts and the London School of Medicine and Dentistry London U.K.; ^8^ St John's Institute of Dermatology Department of Dermatology London U.K.; ^9^ Northern Ireland Cancer Registry Belfast Northern Ireland U.K.; ^10^ School of Medicine University of Dundee Dundee Scotland U.K.

## Abstract

**Background:**

Basal cell carcinoma (BCC) and cutaneous squamous cell carcinoma (cSCC), together known as keratinocyte cancers (KCs), are the commonest cancer in white ethnic populations. Recent improvements to registry data collection in England has allowed more accurate analysis of the epidemiology of BCC and cSCC and for the first time we are able to provide an accurate (representative) tumour burden for KC in the U.K.

**Objectives:**

To estimate the incidence of BCC and cSCC in the U.K.

**Methods:**

A cohort of patients with KCs between 2013 and 2015 were identified using linkage to diagnostic codes derived from pathology reports collected into the national cancer registry. Data from England's cancer registry were combined with data from Scotland, Northern Ireland and Wales. European age‐standardized incidence rates (EASRs) of the first BCC and cSCC per patient per annum (PPPA) were calculated.

**Results:**

In the U.K, the EASR of the first BCC and cSCC PPPA in 2013–15 were 285 and 77 per 100 000 person years, respectively (211 120 KCs total in 2015). The mean annual percentage increase was 5% between 2013 and 2015 for both BCC and cSCC. By counting the first KC PPPA, we include an additional 51% KCs compared with the previous reporting technique which counts only the first BCC and cSCC in a patient's lifetime, yet it represents a probable underestimation of 5–11% of the true tumour count.

**Conclusions:**

Based on an improved methodology, a more representative incidence of KC is presented, which is essential to healthcare planning and will lead to improved understanding of the epidemiology of KC.

**What's already known about this topic?**

- Keratinocyte cancers (KCs) are the most common cancers affecting white ethnic populations.
- The incidence of basal cell carcinoma (BCC) and cutaneous squamous cell carcinoma (cSCC) is increasing worldwide including the U.K., most commonly in elderly male Caucasian patients.
- These cancers are traditionally substantially underreported and frequently excluded from national cancer statistics.

**What does this study add?**

- Using improved data collection methods in England and validated tumour‐reporting techniques, we report the most accurate BCC and cSCC incidence data for the U.K. ever published.
- Identifying the first BCC and cSCC per patient per annum, the incidence of BCC and cSCC in the U.K. (excluding Wales) was 285 and 77 per 100 000 person years, respectively, between 2013 and 2015, with more than 210 000 KCs in the U.K. in 2015.

## Abstract 2

**Linked Editorial**: Whiteman. *Br J Dermatol* 2019; **181**:434–435.

Plain language summary available online

Respond to this article

Keratinocyte cancers (KCs), the collective term for basal cell carcinomas (BCCs) and cutaneous squamous cell carcinomas (cSCCs), are the most common cancers in the U.K. and the most common cancers in white ethnic populations worldwide. However, epidemiological data in the U.K. have historically been of relatively poor quality.^1^, ^2^, ^3^, ^4^, ^5^ Due to high volume and multiplicity of KCs, they have frequently been excluded from national cancer registries and statistics. Previous studies have reported that incidence rates of KCs are increasing worldwide and evidence from local audits suggest that the rate of increase in the U.K. may be higher than in European counterparts.^6^, ^7^, ^8^, ^9^, ^10^, ^11^, ^12^, ^13^ Increasing tumour incidence is presumed to be a result of an ageing population, increased ultraviolet radiation (UVR) exposure with easier access to travel abroad and a higher proportion of fairer skin types in the U.K. compared with other countries,^6^, ^14^ but little is known about the epidemiology of KCs in the U.K.

The healthcare workload burden and cost of KCs is substantial within dermatology departments in the U.K., where these cancers are often seen urgently within 2 weeks of referral. In 2008, skin cancer management costs were estimated to be between £106 and £112 million and approximately £889–1226 per KC in England.^15^ A decade later, these estimates are likely to be substantially higher, with advances in treatment approaches and increase in patient volume. Furthermore, despite a relatively low mortality, morbidity through cosmetic disfigurement and functional morbidity is considerable given that KCs are frequently multiple and most commonly located on the face.

Despite the high volume and associated costs of KCs, limited research progress has been reported. This discrepancy was highlighted by a U.K. Translational Research Network in Dermatology (UKTREND) e‐DELPHI exercise that assessed the research needs of clinicians in the U.K. and identified research into KCs as a priority area.^16^

In 1999, the United Kingdom and Ireland Association of Cancer Registries (UKIACR) ruled that due to complexities in registering multiple pathology reports accurately, only the first BCC or cSCC per patient is reported, despite all pathology reports now being routinely collected since 2013 in England, Scotland and Northern Ireland.^17^ This rule is also used in many cancer registries worldwide.^6^ The impact of this registration approach was that previous figures were clearly significant underestimations of national incidence in the U.K. Metachronous and synchronous BCCs and cSCCs are common.^18^ After a diagnosis of BCC, the 3‐year risk of a subsequent BCC is estimated to be 44%, with 10% developing a further BCC within 6 months.^10^, ^19^, ^20^, ^21^ Likewise, after an initial cSCC, the 5‐year risk of a further cSCC is estimated to be 37%.^21^ Previous studies have shown that when counting all BCCs as opposed to the first registered BCC, an additional 30–50% of tumours are counted.^10^, ^22^, ^23^, ^24^ Changes in cancer registration processes in England, including the introduction of national automated registration have enabled the development of a more comprehensive BCC and cSCC dataset for the first time.

The objective of this study was to validate improved data collection methods and report the incidence and survival for BCC and cSCC from the cancer registry data at a national level for the first time.

## Materials and methods

### Study design, setting and participants

Data were provided by the National Cancer Registration and Analysis Service (NCRAS), England. It is mandatory for all National Health Service pathology laboratories, and recommended to all private pathology laboratories in England, to provide all cancer pathology reports to NCRAS and NCRAS data quality teams to ensure that pathology laboratories are compliant. These pathology reports are combined with information from the Patient Administration System (PAS) and Cancer Outcomes and Services Dataset (COSD) to form a cancer registration record. In England, this process has been nationalized and mostly automated since 2013; a majority of tumours are registered based on the pathology report laboratory codes and text information provided. The minority that do not meet the automated processor's selection criteria are manually registered.

BCCs and cSCCs were identified using the ICD‐10 (International Classification of Diseases) site codes and ICD‐02 morphology and behaviour codes (Table S1, see Supporting Information). The date of receipt of the pathology sample was used as the date of diagnosis, to identify the first BCC and cSCC per patient per annum (PPPA).

Data were also provided by Information Services Division Scotland, the Northern Ireland cancer registry and the Welsh Cancer Intelligence & Surveillance Unit (WCISU). Welsh data collection is acknowledged by the registry to be incomplete (personal communication with WCISU), awaiting changes to be enforced from 2016 data onwards. Welsh data were therefore excluded from the analyses, except for overall tumour count.

### Validation

To confirm the accuracy of the data and the use of the first KC PPPA technique, a randomized selection of 500 patients with BCC and 500 patients with cSCC in 2013 in England were reviewed by a single dermatologist (Z.C.V.). Tumours diagnosed in the previous calendar year, recurrences of previous tumours and incorrectly coded tumours were excluded, e.g. Bowen disease. Additional tumours collected but not counted with the first KC PPPA technique (i.e. more than one tumour per annum) were analysed to provide an estimate of how the first KC PPPA compares with manually registering all tumours per patient and also to assess the validity of the registration process in England. Furthermore, in Scotland, because all cSCCs are registered except in specific cases such as patients with genetic predisposition to multiple cSCCs, Scottish data was also used to assess the first KC PPPA technique vs. registering all tumours manually.

### Variables

Patient demographics such as age (taken as a continuous variable taken from day of diagnosis), sex and self‐reported ethnicity were analysed from NCRAS, which derives information from PAS and COSD. Deprivation quintiles were calculated using the patient's Lower Super Output Area at diagnosis linked to the Index of Multiple Deprivation 2015.^25^

### Statistical analysis

The NCRAS data were extracted using Oracle SQL developer© version 4·1·5·21 (Oracle; Redwood City, CA, U.S.A.). Microsoft© Excel version 2010 (Microsoft; Los Angeles, CA, U.S.A.) and Stata© version 14 (Stata Corporation; College Station, TX, U.S.A.) were used for statistical analyses. Randomization as listed above was performed using a random number generator from Excel© 2010.

European age‐adjusted incidence rates (EASR) are weighted based on European standard populations in 2013 in 5‐year age bands. The EASR of first cSCC and BCC PPPA were calculated across sex and nations (England, Scotland and Northern Ireland). Welsh data were excluded from EASR and age‐specific rates due to lack of completeness. The estimated annual percentage change (EAPC) was calculated as the mean change in EASR per year.

Statistics for absolute number of deaths attributed to nonmelanoma skin cancer was as reported by the Office of National Statistics.^26^

The Pohar Perme estimator was used to calculate age‐standardized net survival with the ‘stns’ command in Stata 14. This analysis was age‐standardized using weights from the International Cancer Survival Standard,^27^ i.e. survival was standardized based on expected survival of age‐ and sex‐specific groups. Life tables were obtained from the Cancer Survival Group at the London School of Hygiene and Tropical Medicine. Vital status of patients was determined until 31 December 2016.^28^

## Results

### Validation

Overall, an additional 51% KC tumours were identified using first KC PPPA technique compared with identifying only the first incident tumour of all time. Analysing the first BCC PPPA in England 2013–15 (*n* = 410 716) compared with first registered (*n* = 268 565) resulted in 53% further BCC tumours recorded over 3 years. Likewise, when counting first cSCC PPPA (*n* = 104 529) compared with first registered (*n* = 76 977) an additional 36% cSCC tumours were recorded.

**Figure 1:**
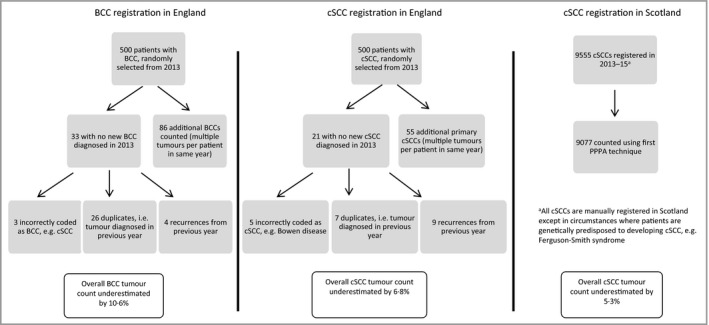
Analysis of first KC per patient per annum (PPPA) technique for counting keratinocyte cancers from registry data. BCC, basal cell carcinoma; cSCC, cutaneous squamous cell carcinoma.

To assess the first KC PPPA technique and use of an automated processor in England, a randomly selected cohort of 500 patients with BCC and 500 with cSCC from the English cancer registry 2013 were selected and the number of tumours per patient was counted. Counting the first KC PPPA resulted in 10·6% fewer BCC tumours and 6·8% fewer cSCC tumours (Fig. 1).

In Scotland, all cSCC tumours are registered manually except in cases of genetic predisposition; therefore, we compared the registered cSCC tumour count with the first KC PPPA technique in Scotland. This found an underestimation by 5·3% of all cSCCs by using the first KC PPPA (Fig. 1).

Analysing the automated processing technique in England, only 0·8% (8 of 1000) tumours were incorrectly coded, e.g. Bowen disease coded as an invasive cSCC.

### U.K. incidence of keratinocyte cancers

**Figure 2:**
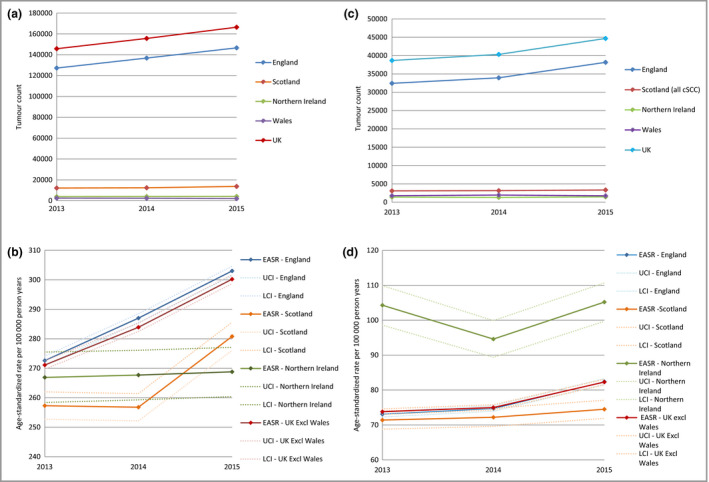
(a–d) National tumour count and incidence rate of BCC and cSCC using the first tumour per patient per annum technique except for cSCC data for Scotland where all cSCCs are registered; Welsh data are excluded from incidence rates due to incomplete data collection. (a) National BCC tumour count 2013–15. (b) National EASR of BCCs 2013–15. (c) National cSCC tumour count 2013–15. (d) National EASR of cSCCs 2013–15. EASR, European age‐standardized rate; BCC, basal cell carcinoma; cSCC, cutaneous squamous cell carcinoma; LCI, lower 95% confidence interval; UCI, upper 95% confidence interval.

The absolute first BCC PPPA count increased in the 3‐year period from 145 817 to 166 448 in 2015 (Fig. 2a). The EASR of first BCC PPPA in the U.K. from 2013 to 2015 was 352 per 100 000 person years (PY) in males and 219 per 100 000 PY in females. More than 85% of BCCs in the U.K. occurred in England and the EASR was highest in England compared with other U.K. nations (Fig. 2b). For the U.K., the EAPC was 5% for BCC, but much lower in Northern Ireland (0·3%).

The absolute count of first cSCC PPPA increased from 38 664 in 2013 to 44 672 in 2015 in the U.K. (Fig. 2c). The EASR of first cSCC PPPA from 2013 to 2015 was 111 in men and 42 per 100 000 PY in women with the highest rates seen in Northern Ireland (Fig. 2d). The EAPC of cSCC was around 6% in the 3‐year period.

### Geographic incidence differences in the U.K

**Figure 3:**
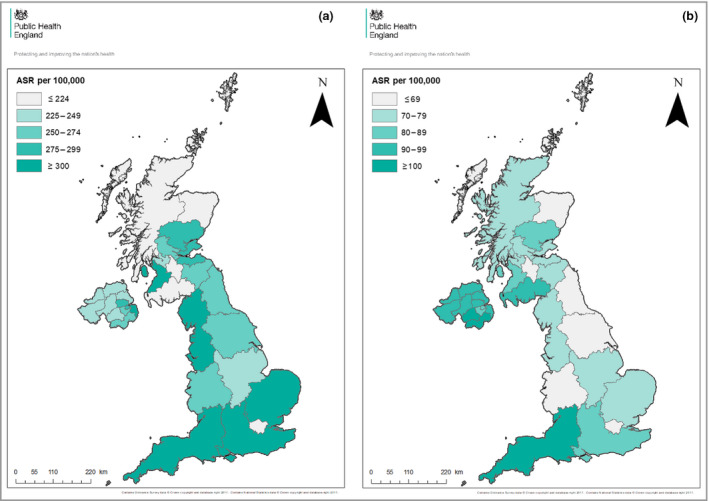
(a) Regional BCC EASRs in the U.K. 2013–15 using the first KC PPPA technique. Welsh data are excluded due to incomplete data collection. (b) Regional cSCC EASR in the U.K. 2013–15 using the first KC PPPA technique except in Scotland where all cSCC are registered. Welsh data are excluded due to incomplete data collection. EASR, European age‐standardized incidence rates; BCC, basal cell carcinoma; cSCC, cutaneous squamous cell carcinoma; PPPA, per patient per annum.

The incidence rates of first KC PPPA differ across the U.K. and are highest in southern and coastal regions (Fig. 3). The highest BCC rates were observed in Southwest England (EASR 362 per 100 000 PY) and the lowest EASR was in Dumfries and Galloway (39 per 100 000 PY). For cSCC, the highest rates were observed in Southwest England and the lowest in Shetland (EASR 107 and 345 per 100 000 PY, respectively). Several regions such as London and Northern Scotland have notably lower EASR as expected.

### Demographics of patients with keratinocyte cancers in England

**Figure 4:**
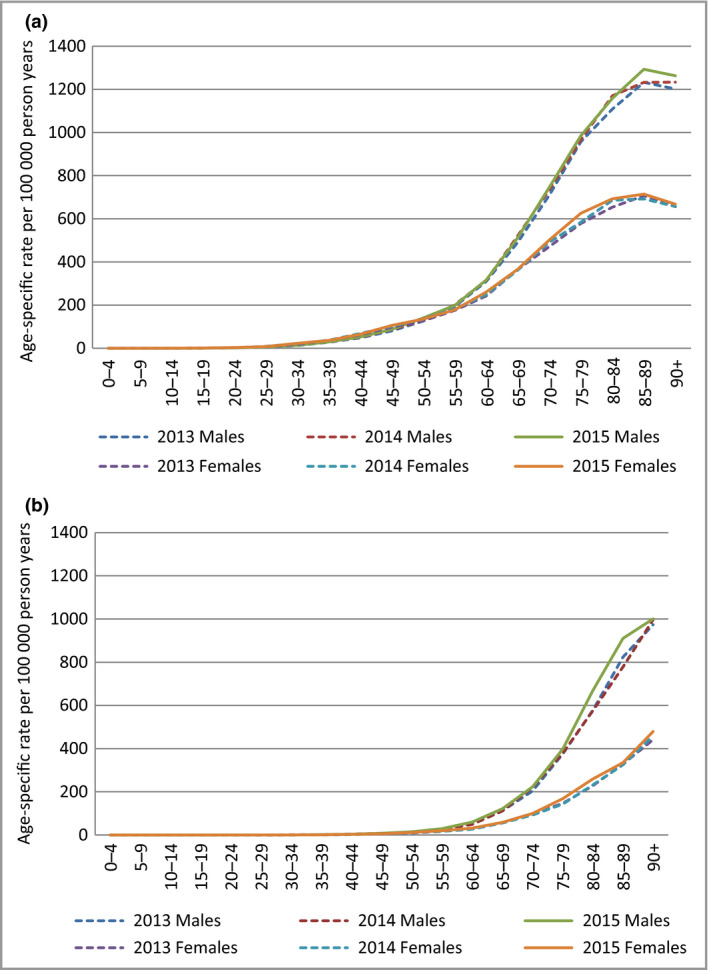
(a–b) Age‐specific rates of basal cell carcinoma (BCC) and cutaneous squamous cell carcinoma (cSCC) in males and females 2013–15, England. Using data for first tumour all time. (a) Age‐specific rates of BCC. (b) Age‐specific rates of cSCC.

Patients with first all‐time BCC and cSCC were more commonly males, with a male : female ratio 1·2 : 1 for BCC and 1·7 : 1 for cSCC (Table 1). The median age at time of incident tumour was approximately 71 years [interquartile range (IQR) = 62–80] for BCC and 79 years (IQR 71–85) for cSCC. The age‐specific rates for both BCC and cSCC clearly increase with age and are much higher and steeper among men than women (Fig. 4). For both BCC and cSCC, fewer than 1% of individuals self‐reported as nonwhite, with a substantial proportion of missing data (up to 34%). The distribution of the deprivation quintiles is comparable for both tumour types between men and women.

**Table 1:** Patient demographics of first all‐time registered BCC and cSCC, England 2013–15

Patient demographics of first registered tumour	First BCC, *n* = 268 565		First cSCC *n* = 76 977	
	Male, *n* = 144 680	Female, *n* = 123 885	Male, *n* = 48 254	Female, *n* = 28 723
Age (years)				
Median (IQR)	72 (63–79)	71 (60–80)	78 (71–84)	80 (71–87)
0–29	464 (0·3)	641 (0·5)	30 (0·1)	26 (0·1)
30–39	2229 (1·5)	3066 (2·5)	123 (0·3)	98 (0·3)
40–49	7685 (5·3)	9680 (7·8)	655 (1·4)	489 (1·7)
50–59	16 660 (11·5)	16 148 (13·0)	2117 (4·4)	1491 (5·2)
60–69	35 487 (24·5)	27 893 (22·5)	7352 (15·2)	3930 (13·7)
70–79	46 551 (32·2)	33 990 (27·4)	16 158 (33·5)	7735 (26·9)
80–89	30 631 (21·2)	25 913 (20·9)	17 822 (36·9)	10 385 (36·2)
90+	4973 (3·4)	6554 (5·3)	3997 (8·3)	4569 (15·9)
Ethnicity				
White	97 418 (67·3)	80 690 (65·1)	43 283 (89·7)	25 226 (87·8)
Mixed	63 (0·0)	69 (0·1)	29 (0·1)	22 (0·1)
Indian /other Asian background	108 (0·1)	114 (0·1)	73 (0·2)	50 (0·2)
Afro‐Caribbean/other black background	57 (0·0)	61 (0·0)	42 (0·1)	38 (0·1)
Chinese	22 (0·0)	23 (0·0)	7 (0·0)	8 (0·0)
Other ethnic group	402 (0·3)	371 (0·3)	169 (0·4)	96 (0·3)
Unknown	46 610 (32·2)	42 557 (34·4)	4651 (9·6)	3283 (11·4)
Deprivation quintiles				
1 (least deprived)	40 366 (27·9)	32 572 (26·3)	12 792 (26·5)	7096 (24·7)
2	37 363 (25·8)	31 004 (25·0)	12 451 (25·8)	7064 (24·6)
3	30 101 (20·8)	26 108 (21·1)	10 200 (21·1)	6295 (21·9)
4	21 915 (15·1)	20 045 (16·2)	7642 (15·8)	4825 (16·8)
5 (most deprived)	14 935 (10·3)	14 156 (11·4)	5169 (10·7)	3443 (12·0)
Site of first registered BCC or cSCC				
Lip (cutaneous)	1491 (1·0)	3219 (2·6)	815 (1·7)	679 (2·4)
Eyelid incl. canthus	6353 (4·4)	8019 (6·5)	584 (1·2)	483 (1·7)
Ear	9439 (6·5)	1568 (1·3)	7601 (15·8)	363 (1·3)
Face	55 213 (38·2)	50 691 (40·9)	13 126 (27·2)	9308 (32·4)
Scalp/neck	11 315 (7·8)	8265 (6·7)	11 566 (24·0)	1669 (5·8)
Trunk incl. perianal	22 630 (15·6)	14 484 (11·7)	3080 (6·4)	2201 (7·7)
Upper limb incl. shoulder	9870 (6·8)	7479 (6·0)	6947 (14·4)	5416 (18·9)
Lower limb incl. hip	5637 (3·9)	10 951 (8·8)	2367 (4·9)	7205 (25·1)
Skin NOS	22 732 (15·7)	19 209 (15·5)	2168 (4·5)	1399 (4·9)

Data are *n* (%) unless otherwise specified. BCC, basal cell carcinoma; CI, confidence interval; cSCC, cutaneous squamous cell carcinoma; IQR, interquartile range; NOS, not otherwise specified

### Body site of keratinocyte cancers in England

The majority of KCs are located in the head and neck region, especially on the face (between 27% and 40·9%; Table 1). Compared with cSCCs, BCCs tend to be more common on the trunk and are less frequent on the upper extremities. In men, 15·8% of cSCCs were located on the ear whereas the ear was affected in only 1·3% of women. A quarter of cSCCs in women were located on the lower extremities, which is five‐fold more than in men. In approximately 15% of BCCs and 5% of cSCCs, body site was not reported (Table 1).

### Keratinocyte cancer‐specific mortality in England

The absolute number of deaths with an underlying cause of nonmelanoma skin cancers (which includes other skin cancers such as Merkel cell carcinomas) in England were 489 in 2013, 626 in 2014 and 624 in 2015.

Three‐year net survival was 101·9% [95% confidence interval (CI) 101·8–102·0] for BCC and 96·2% (95% CI 95·9–96·6) for cSCC overall. For men, 3‐year net survival was 101·9% (95% CI 101·8–102·1) for BCC and 95·7% (95% CI 95·3–96·2) for cSCC. For women, 3‐year net survival was 101·9% (95% CI 101·7–102·0) for BCC and 97·1% (96% CI 96·6–97·6) for cSCC.

## Discussion

We report the largest and most complete dataset for national incidence of KCs ever published, with over 210 000 tumours reported in 2015 in the U.K. With significant increases in tumour count between 2013 and 2015, KCs represent an overwhelming burden on the workload of healthcare resources. KCs occur mainly in older people, and because of steadily ageing populations, the pressure on the health service is likely to further increase. Of note, skin cancers were four times more common than any other cancer in the U.K. in 2015.^26^ This study provides much needed information, as highlighted in recent epidemiological reviews of KC.^3^, ^7^, ^8^, ^9^, ^29^

Due to the frequent multiplicity of KCs, the UKIACR method of counting only one tumour per patient has resulted in substantial underestimation of national tumour counts. We validate a technique to assess the first BCC and cSCC PPPA, which identified 51% additional tumours compared with the standard first registered BCC and cSCC UKIACR method. While less accurate than manually registering all KCs, this method allows improved incidence reports with minimal additional workload and is easily achievable with access to pathology or cancer registry data. Our validation study would imply that when counting all tumours, i.e. multiple tumours per patient per year, the true tumour count is likely to be 5–11% higher than those provided by the first KC PPPA technique.

There are strengths and limitations to our study. The combined U.K. registries form the largest population‐based KC registry in the world, an essential tool for assessing the epidemiology of these tumours and the main strength of our study. However, inherent to large national pathology‐based cancer registries there are several potential sources of underreporting such as use of topical and destructive treatments without histological confirmation, miscoding or the impact of long waiting lists. Also, there are regional differences in KC registration within the U.K. that may have affected comparisons between regions. For example, all cSCCs are manually registered in Scotland; therefore, rates will be comparatively higher than first KC PPPA counts; despite this, rates remain lower than in England and Northern Ireland. Due to the introduction of new automated report‐processing technology in 2013, the data collection prior to 2013 may be less complete. Therefore, the increase in first registered tumours in subsequent years may be an overestimation, but this does not apply to first KC PPPA reporting. Because the KC registry is based on routinely collected data and linked to other national data sources, the granularity of the data is not always sufficient, e.g. missing data in self‐reported ethnicity, tumour localization and lack of UVR exposure data.

Primary cSCCs affecting perianal sites have a different pathogenesis, with human papillomavirus infection thought to be an important cause rather than UVR.^30^ However, ICD‐10 coding of perianal tumours classifies these to be coded as ‘truncal’ tumours and therefore this precludes accurate identification of these tumours.

Regarding interpretation, the distribution of recorded anatomical sites for primary KCs differed between men and women, which is in line with other studies.^4^, ^20^ This may relate to varying UVR exposure as a result of male‐pattern baldness and cultural preferences, i.e. shorter hair for men and women wearing dresses/skirts. This may explain why men are more likely to develop KCs on the ear and scalp, and for women the lower limb is preferentially affected. However, the commonest site in both sexes for KC was the face.

The regional variations identified in KC incidence may reflect reducing incidence with higher latitude/lower UVR exposure. In addition, in urban areas ethnicity may be more diverse, and behaviour may differ in terms of outdoor work and activities (Fig. 3). It is unclear why Northern Ireland has a higher cSCC incidence compared with other nations; this could relate to a higher number of outdoor occupations, recent public health campaigns, variation in clinical practice or data collection.^31^, ^32^ Compared with elsewhere in the world, incidence rates of KC in the U.K. are lower than those published in Australia, but higher than elsewhere in Europe and the U.S.A., with varying rates presumably due to skin type/genetic predisposition and UVR exposure.^6^, ^10^, ^18^, ^24^

Similar to previous studies, we show that BCC and cSCC are both significantly associated with lower deprivation quintiles.^33^ This is likely to be the result of the expense of foreign travel and increased leisure time being more affordable in these quintiles, equating to higher UVR exposure in the generations affected as well as increased awareness of skin cancer and access to health care.

According to national statistics, 86% of the population in England and Wales are of ‘white’ ethnicity; however, of those with known ethnicity, 99·3% of patients with primary cSCC are of white ethnicity, which may be mainly due to these skin types having reduced protection from UVR.^34^

Three year net survival of KC is over 95% for both types and the overall prognosis of KC is very good with only a minority of patients with cSCC developing advanced disease. Despite low mortality rates, KCs represent an overwhelming tumour burden in the population.^35^ We confirm an interesting observation that patients who develop their first BCC have a 3‐year net survival of over 100%.^36^ This can be in part the result of the association of BCC with lower deprivation quintiles, fitness for biopsy excluding older more frail patients from our cohort and general adherence to healthier outdoor lifestyles. Figure 4 shows that at the age of 85 years a peak in incidence rate occurs, suggesting that patients are excluded where the risk outweighs the benefit for BCC treatment, i.e. frailer, older patients with more comorbidities.

In conclusion, a new technique taking advantage of the modernization of national cancer registration and data collection in the U.K. has resulted in significantly improved reporting of KC incidence. We have validated this registration and data collection approach here and now demonstrate more accurately the huge and steadily increasing trend in KC incidence in the U.K. The scale of the disease burden posed by KC inevitably has implications for healthcare resources. The more accurate incidence data reported here, and now available prospectively through improved KC registration, will undoubtedly facilitate improved service planning and provision in the future. In addition, it will lead to improved understanding of the natural history and prognosis of KC. Finally, it provides further evidence for the importance of future skin cancer prevention initiatives as part of strategies to reduce the morbidity and future healthcare resource implications of KC.

## Supporting information


**Table S1** Classification of basal cell carcinoma and cutaneous squamous cell carcinoma.

**Powerpoint S1** Journal Club Slide Set.
