# Supplementary material for: Epidemiology of basal and cutaneous squamous cell carcinoma in the U.K. 2013–15: a cohort study
Source: Br J Dermatol. 2019 May 6;181(3):474–82. doi: 10.1111/bjd.17873 (PMC7379277; doi:10.1111/bjd.17873)
Supplement: Supplementary file 1 — Table S1 Classification of basal cell carcinoma and cutaneous squamous cell carcinoma. [file BJD-181-474-s001.docx]

|  | ICD-10 site code | ICD-02 morphology code | ICD-02 behaviour code |
| --- | --- | --- | --- |
| BCC | C44* | 8090-8095 and 8097 | 3** |
| cSCC | C44* | 8050-8052, 8070- 8078, 8082- 8084 | 3** |

*C44 =Non melanoma skin cancer
**Behaviour code 3 = malignant

Supplementary Table. Classification of BCC and cSCC
