# Supplementary material for: Epidemiology of basal and cutaneous squamous cell carcinoma in the U.K. 2013–15: a cohort study
Source: Br J Dermatol. 2019 May 6;181(3):474–82. doi: 10.1111/bjd.17873 (PMC7379277; doi:10.1111/bjd.17873)
Supplement: Supplementary file 2 — Powerpoint S1 Journal Club Slide Set. [file BJD-181-474-s002.pptx]

## Slide 1
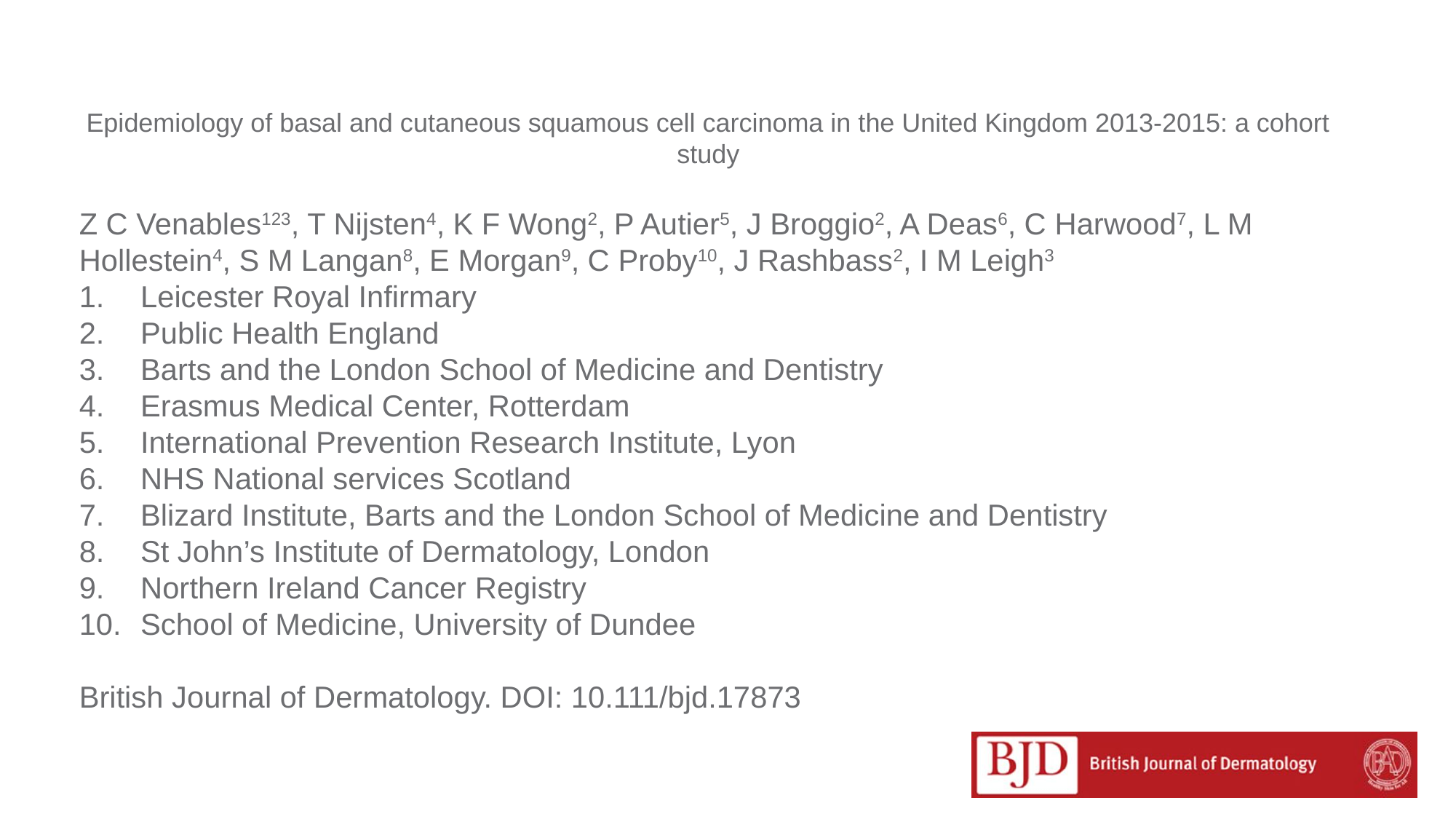

# Epidemiology of basal and cutaneous squamous cell carcinoma in the United Kingdom 2013-2015: a cohort study
Z C Venables123, T Nijsten4, K F Wong2, P Autier5, J Broggio2, A Deas6, C Harwood7, L M Hollestein4, S M Langan8, E Morgan9, C Proby10, J Rashbass2, I M Leigh3
Leicester Royal Infirmary
Public Health England
Barts and the London School of Medicine and Dentistry
Erasmus Medical Center, Rotterdam
International Prevention Research Institute, Lyon
NHS National services Scotland
Blizard Institute, Barts and the London School of Medicine and Dentistry
St John’s Institute of Dermatology, London
Northern Ireland Cancer Registry
School of Medicine, University of Dundee
British Journal of Dermatology. DOI: 10.111/bjd.17873

## Slide 2
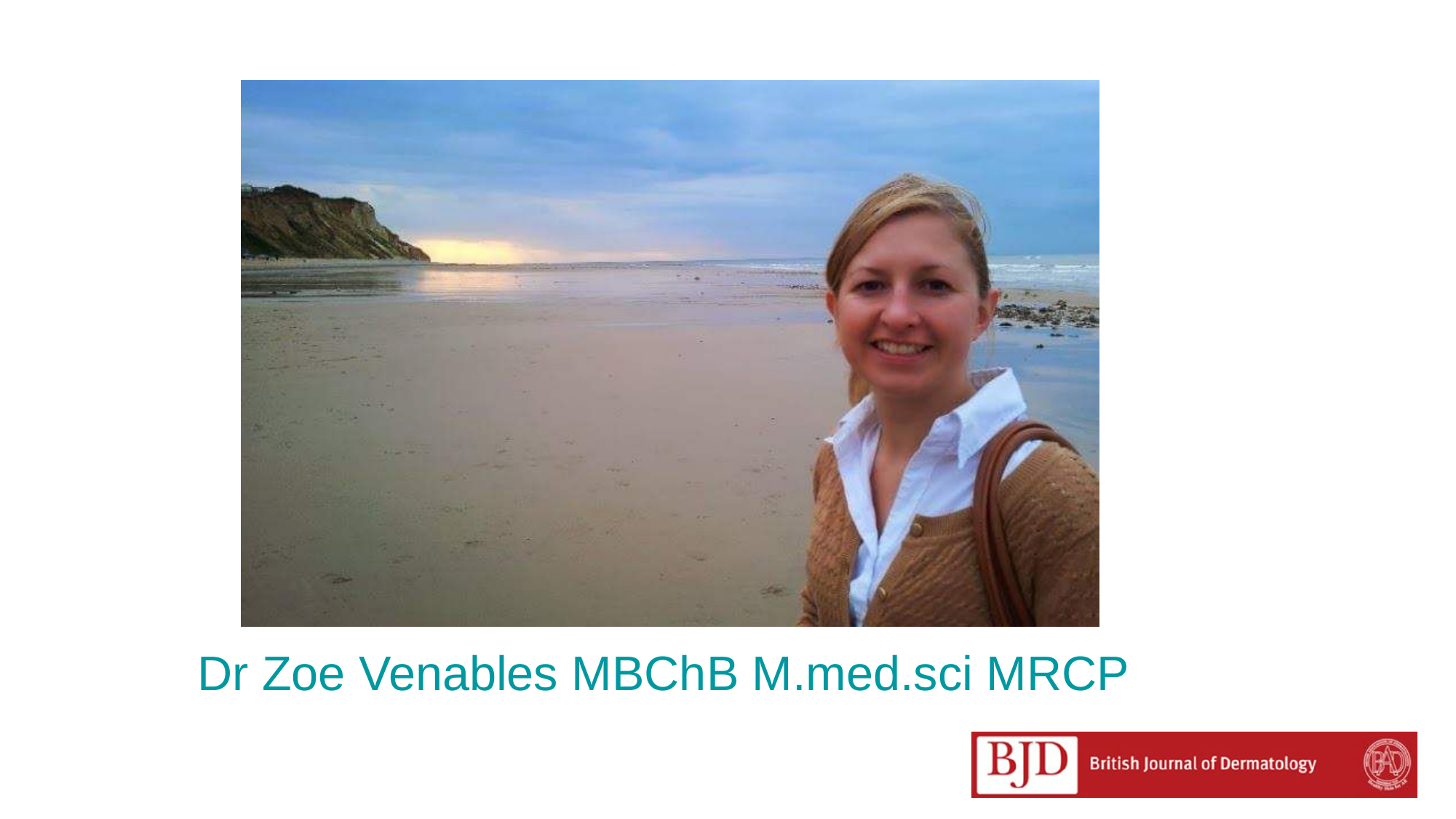

# Dr Zoe Venables MBChB M.med.sci MRCP

## Slide 3
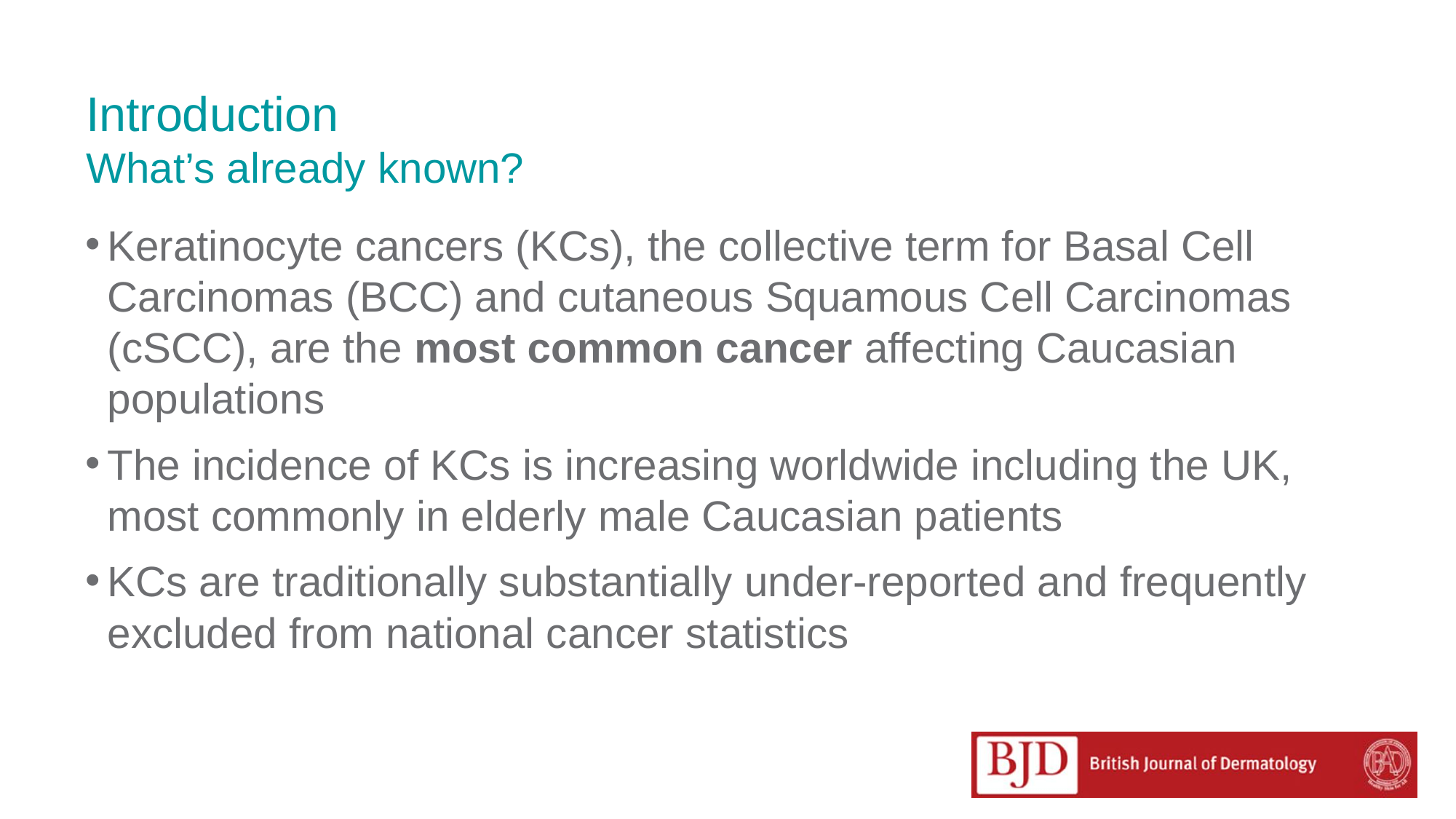

# Introduction What’s already known?
Keratinocyte cancers (KCs), the collective term for Basal Cell Carcinomas (BCC) and cutaneous Squamous Cell Carcinomas (cSCC), are the most common cancer affecting Caucasian populations
The incidence of KCs is increasing worldwide including the UK, most commonly in elderly male Caucasian patients
KCs are traditionally substantially under-reported and frequently excluded from national cancer statistics

## Slide 4
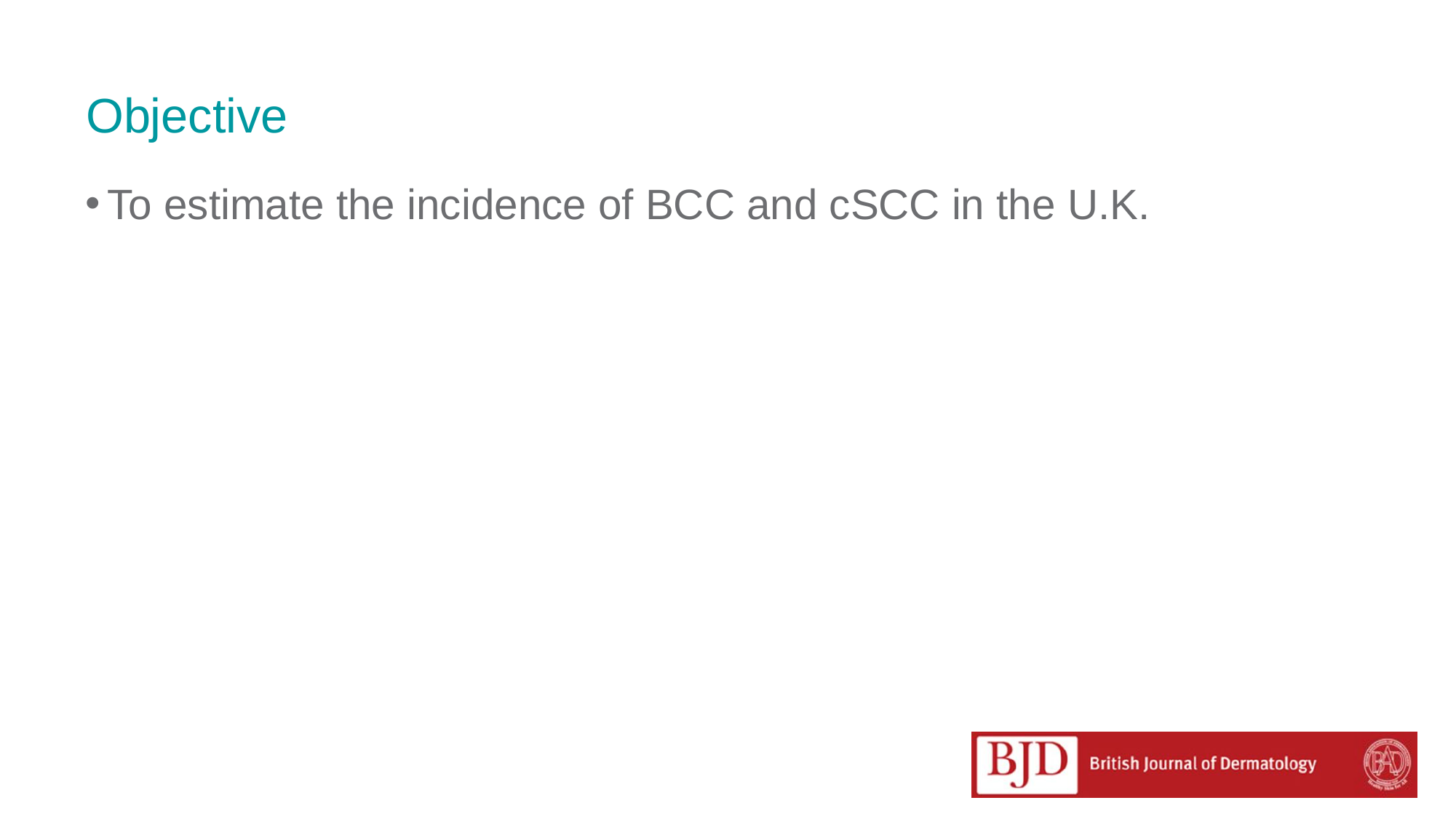

# Objective
To estimate the incidence of BCC and cSCC in the U.K.

## Slide 5
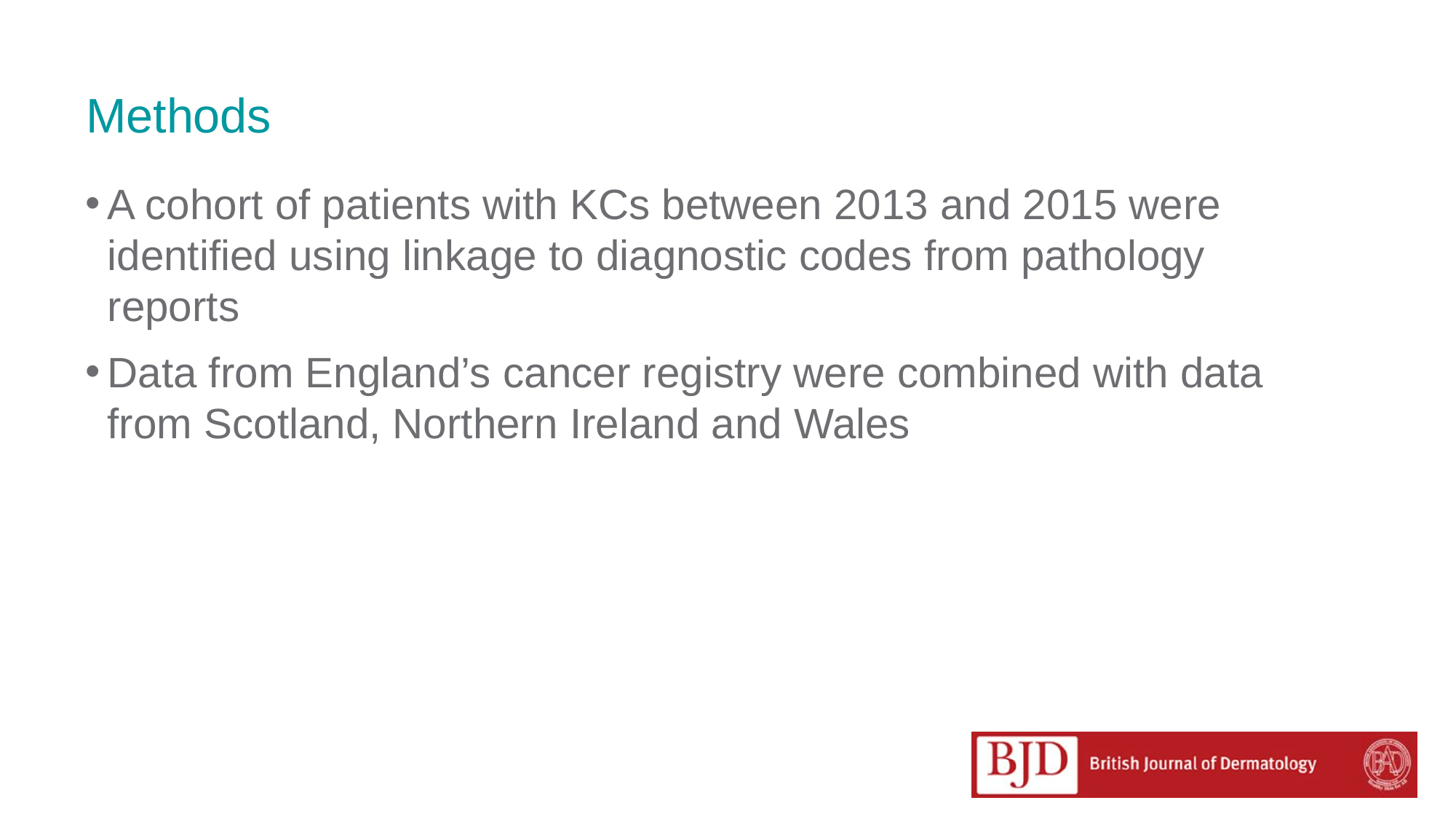

# Methods
A cohort of patients with KCs between 2013 and 2015 were identified using linkage to diagnostic codes from pathology reports
Data from England’s cancer registry were combined with data from Scotland, Northern Ireland and Wales

## Slide 6
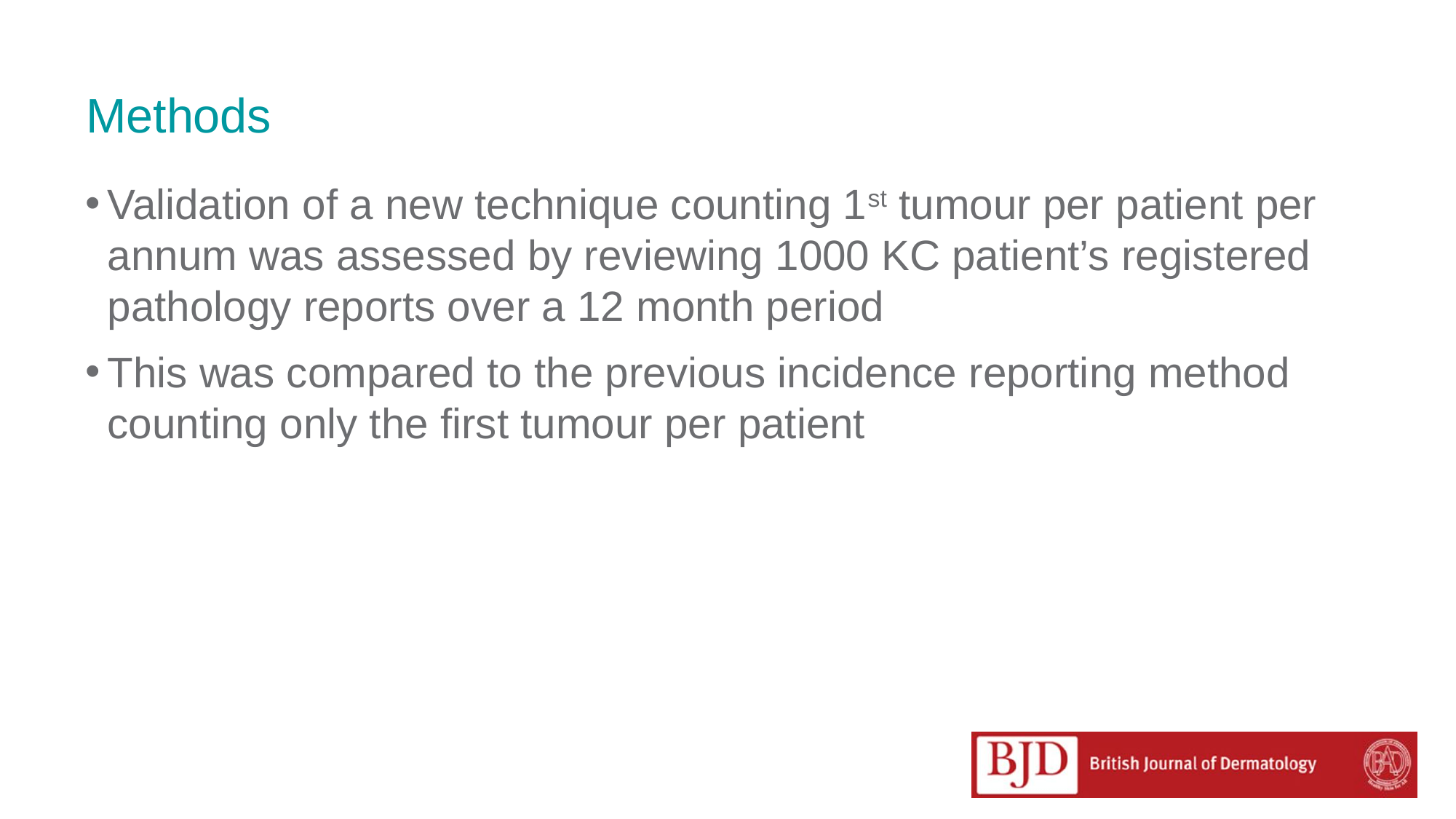

# Methods
Validation of a new technique counting 1st tumour per patient per annum was assessed by reviewing 1000 KC patient’s registered pathology reports over a 12 month period
This was compared to the previous incidence reporting method counting only the first tumour per patient

## Slide 7
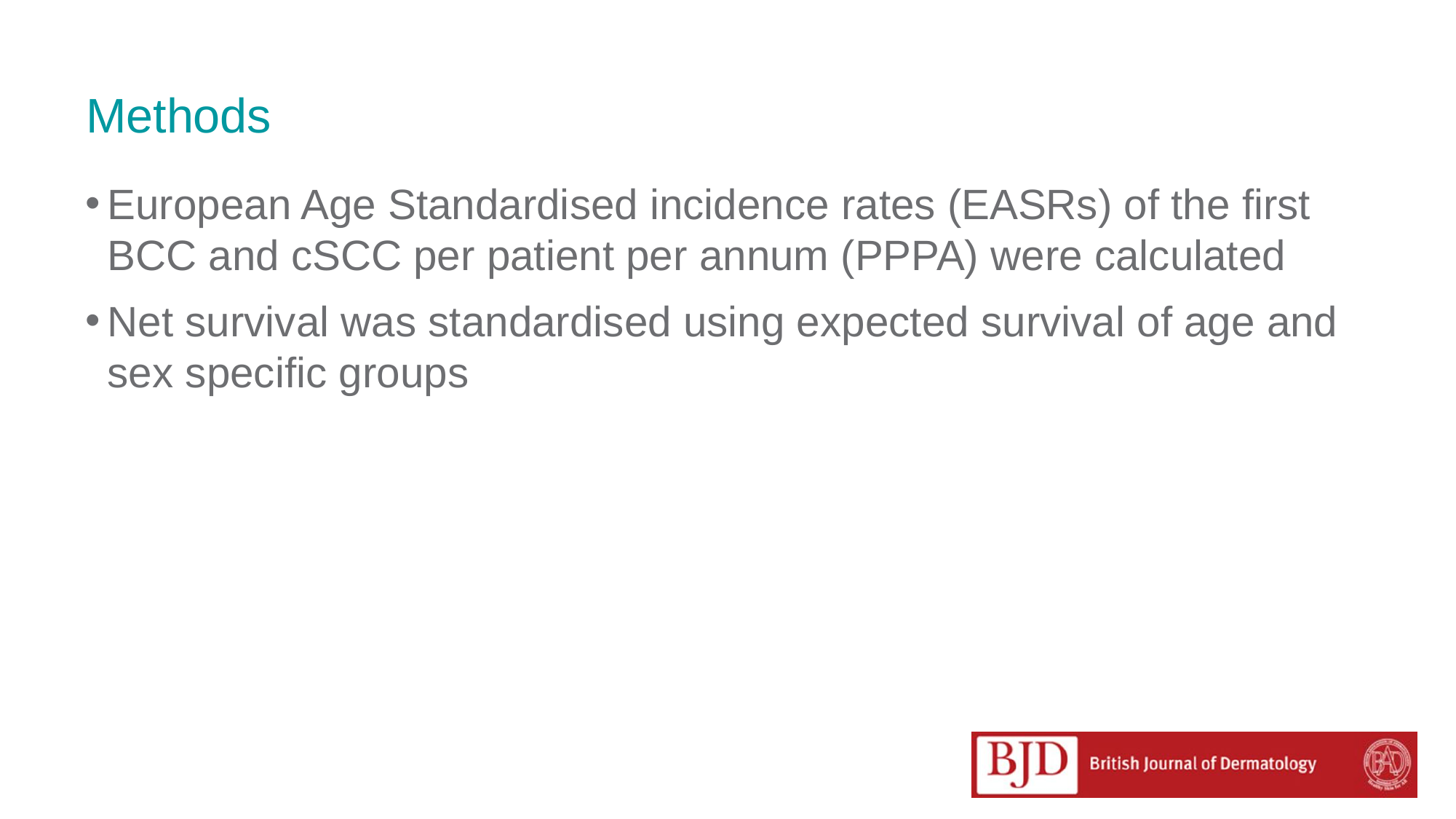

# Methods
European Age Standardised incidence rates (EASRs) of the first BCC and cSCC per patient per annum (PPPA) were calculated
Net survival was standardised using expected survival of age and sex specific groups

## Slide 8
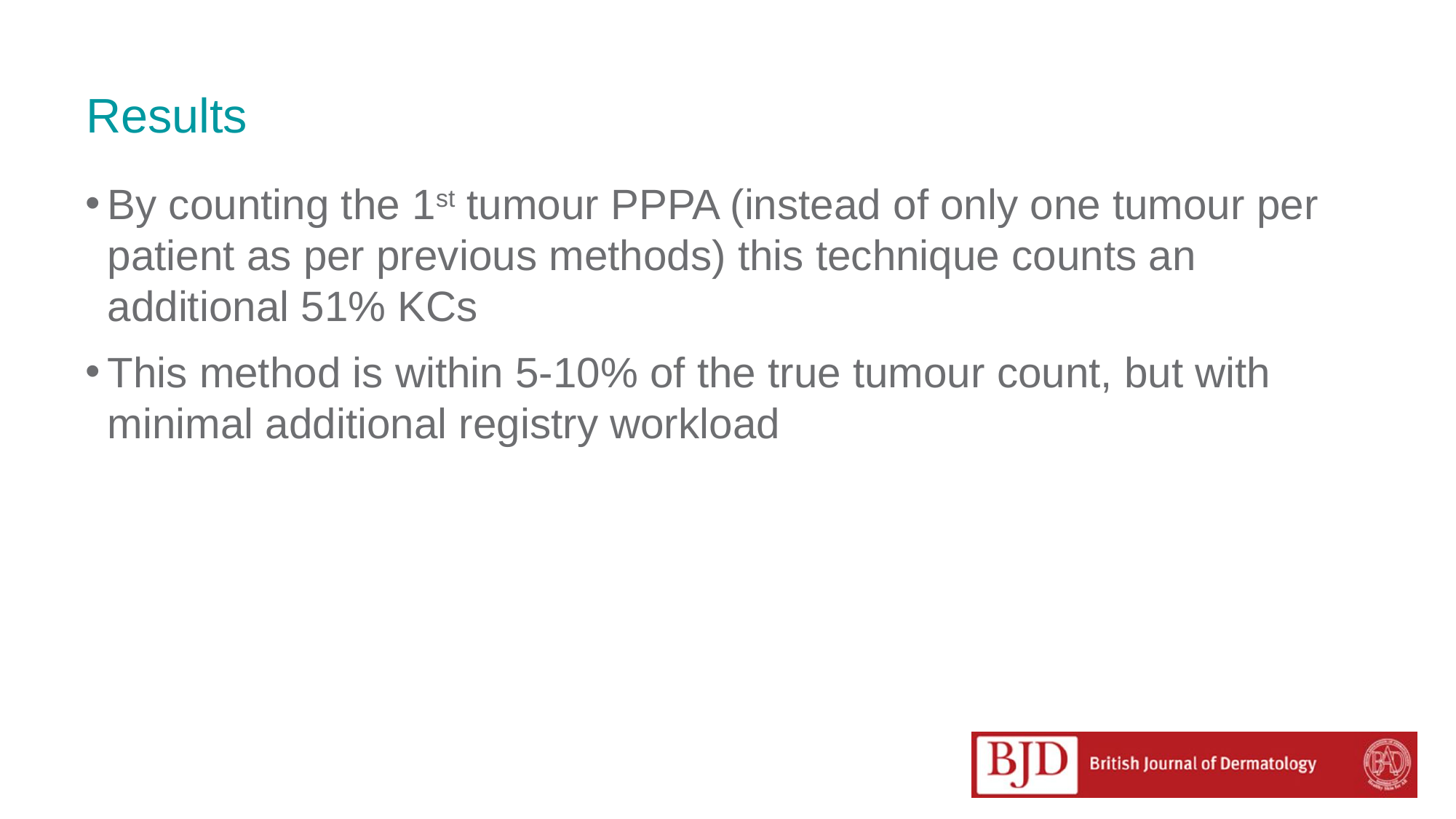

# Results
By counting the 1st tumour PPPA (instead of only one tumour per patient as per previous methods) this technique counts an additional 51% KCs
This method is within 5-10% of the true tumour count, but with minimal additional registry workload

## Slide 9
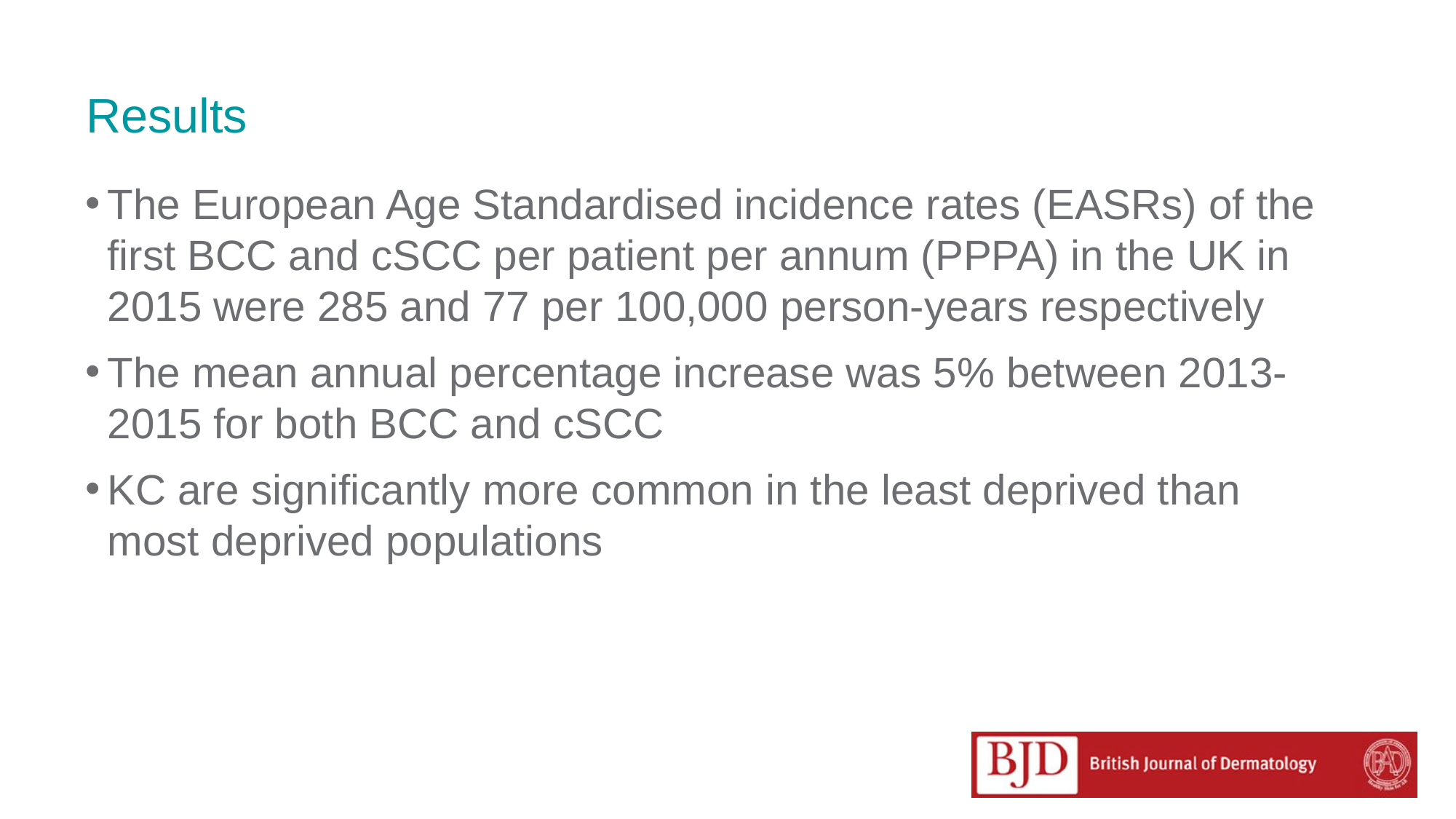

# Results
The European Age Standardised incidence rates (EASRs) of the first BCC and cSCC per patient per annum (PPPA) in the UK in 2015 were 285 and 77 per 100,000 person-years respectively
The mean annual percentage increase was 5% between 2013-2015 for both BCC and cSCC
KC are significantly more common in the least deprived than most deprived populations

## Slide 10
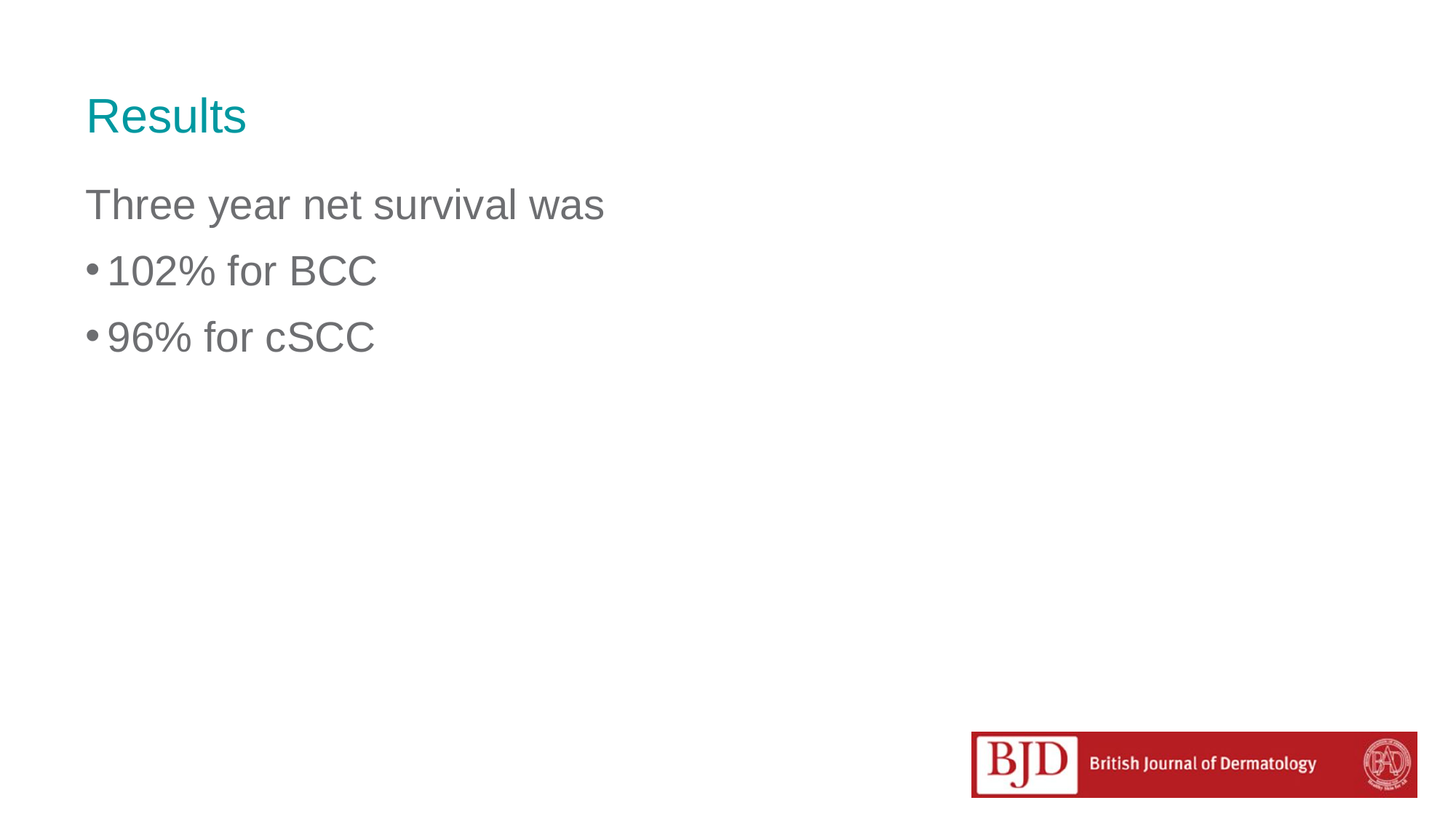

# Results
Three year net survival was
102% for BCC
96% for cSCC

## Slide 11
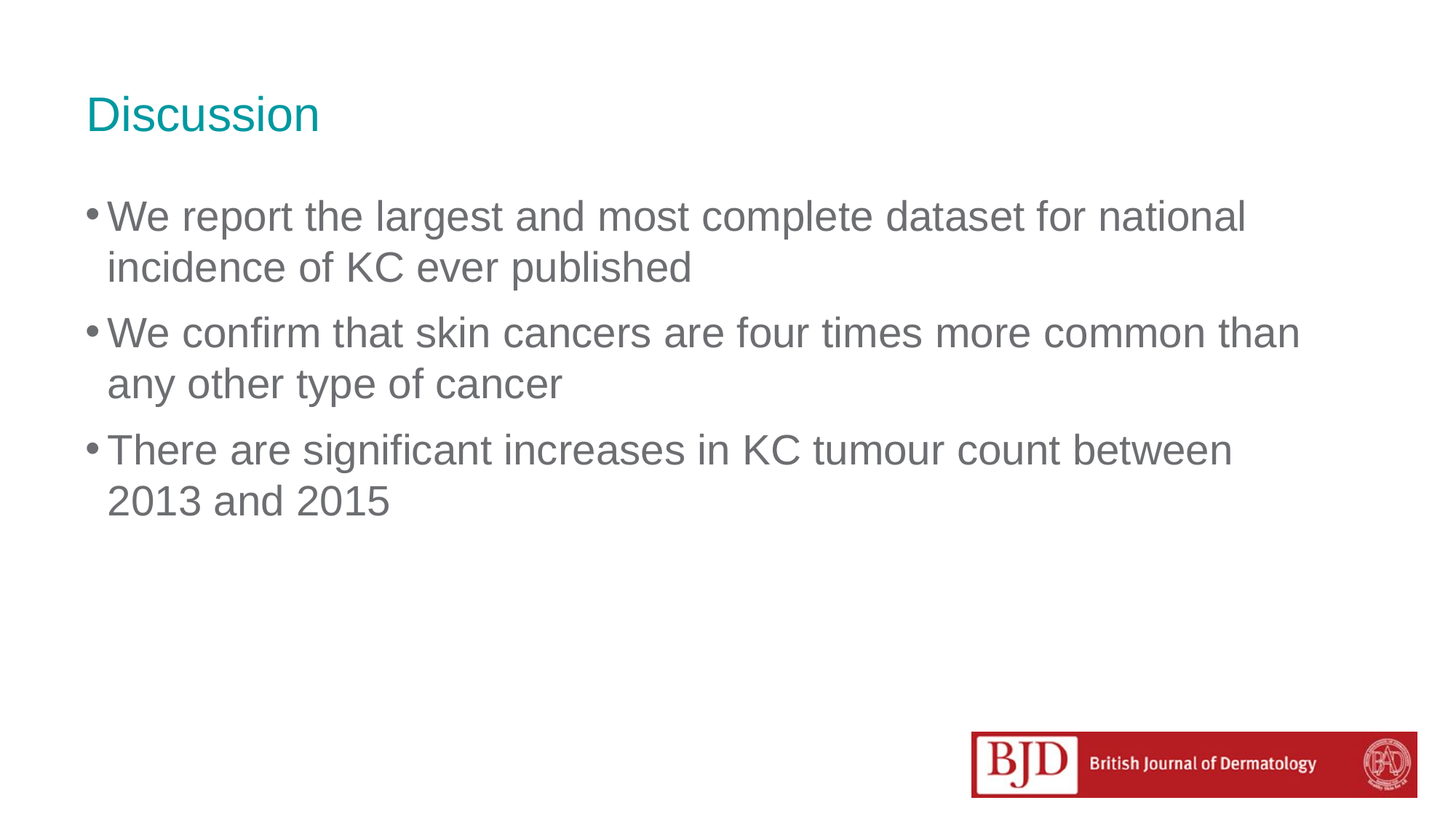

# Discussion
We report the largest and most complete dataset for national incidence of KC ever published
We confirm that skin cancers are four times more common than any other type of cancer
There are significant increases in KC tumour count between 2013 and 2015

## Slide 12
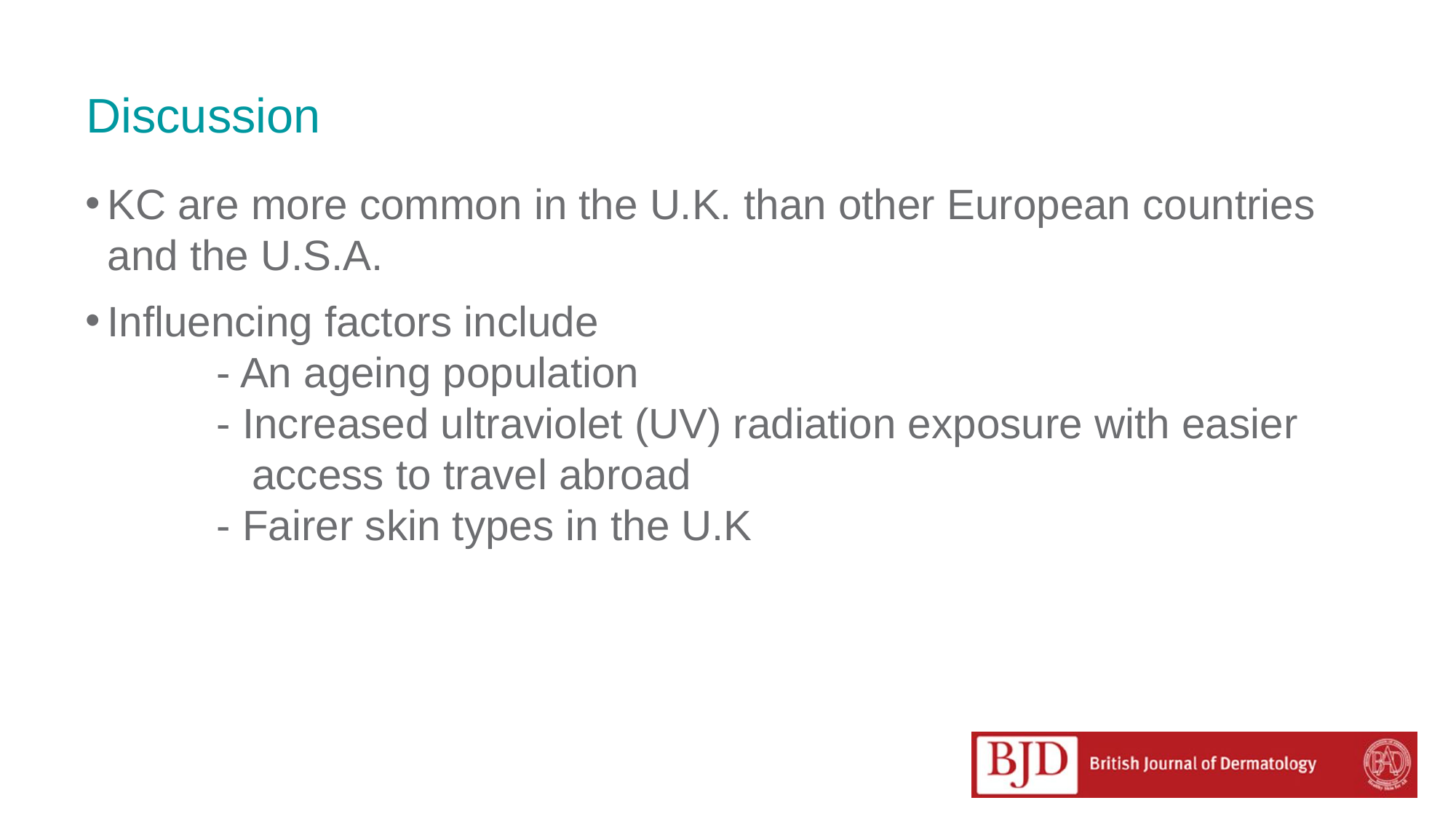

# Discussion
KC are more common in the U.K. than other European countries and the U.S.A.
Influencing factors include
	- An ageing population
	- Increased ultraviolet (UV) radiation exposure with easier 	 access to travel abroad
	- Fairer skin types in the U.K

## Slide 13
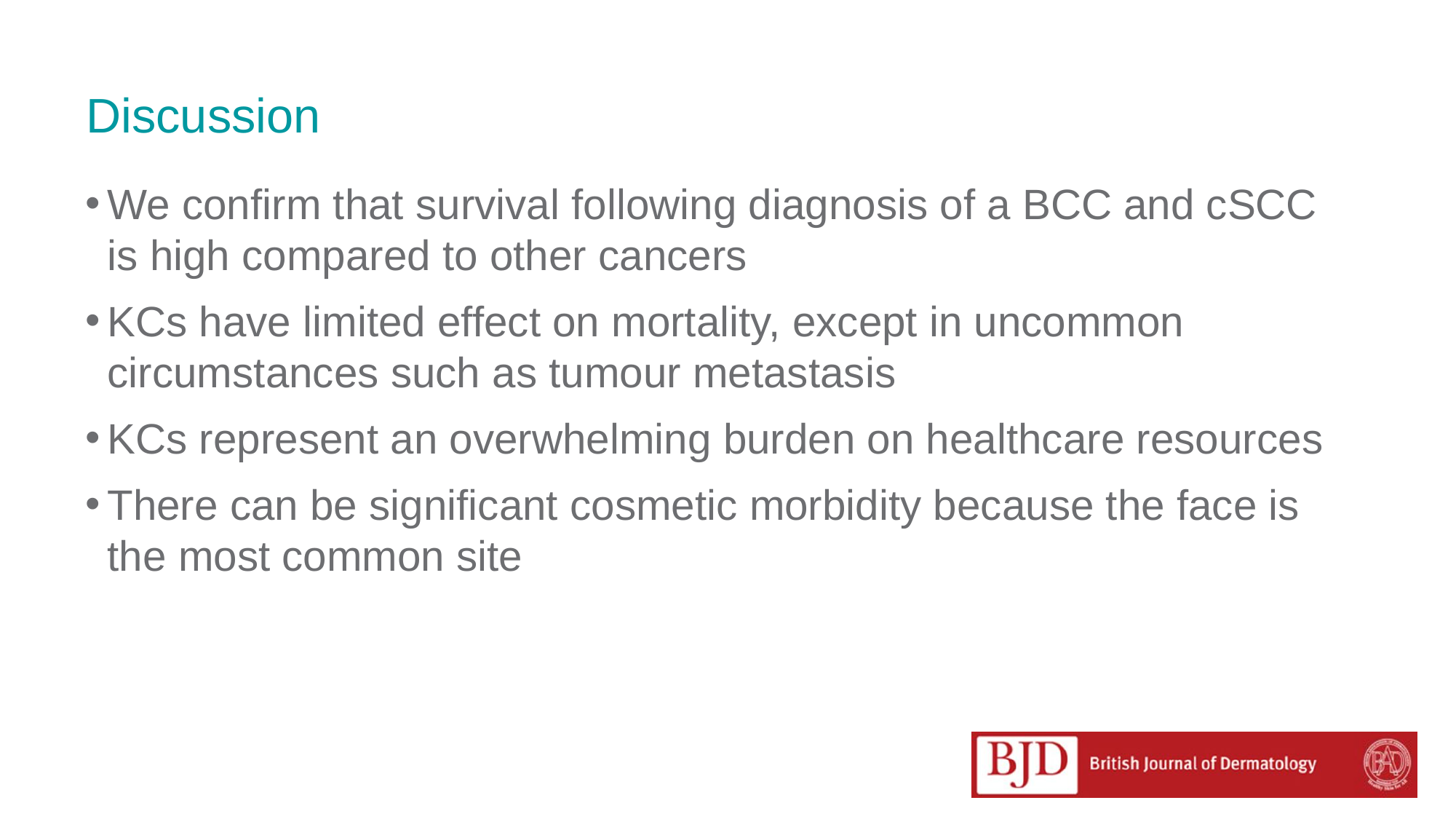

# Discussion
We confirm that survival following diagnosis of a BCC and cSCC is high compared to other cancers
KCs have limited effect on mortality, except in uncommon circumstances such as tumour metastasis
KCs represent an overwhelming burden on healthcare resources
There can be significant cosmetic morbidity because the face is the most common site

## Slide 14
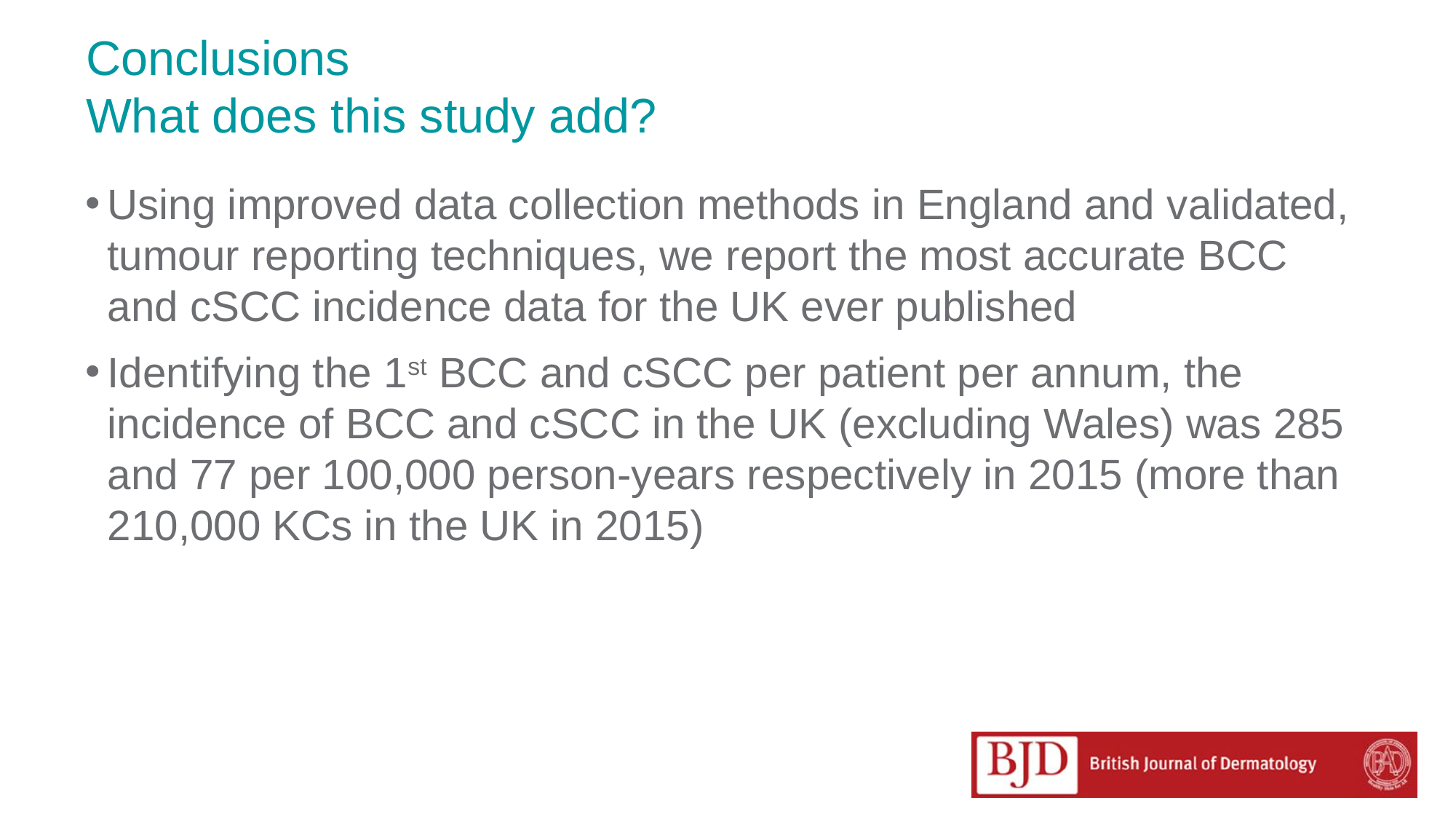

# Conclusions What does this study add?
Using improved data collection methods in England and validated, tumour reporting techniques, we report the most accurate BCC and cSCC incidence data for the UK ever published
Identifying the 1st BCC and cSCC per patient per annum, the incidence of BCC and cSCC in the UK (excluding Wales) was 285 and 77 per 100,000 person-years respectively in 2015 (more than 210,000 KCs in the UK in 2015)

## Slide 15
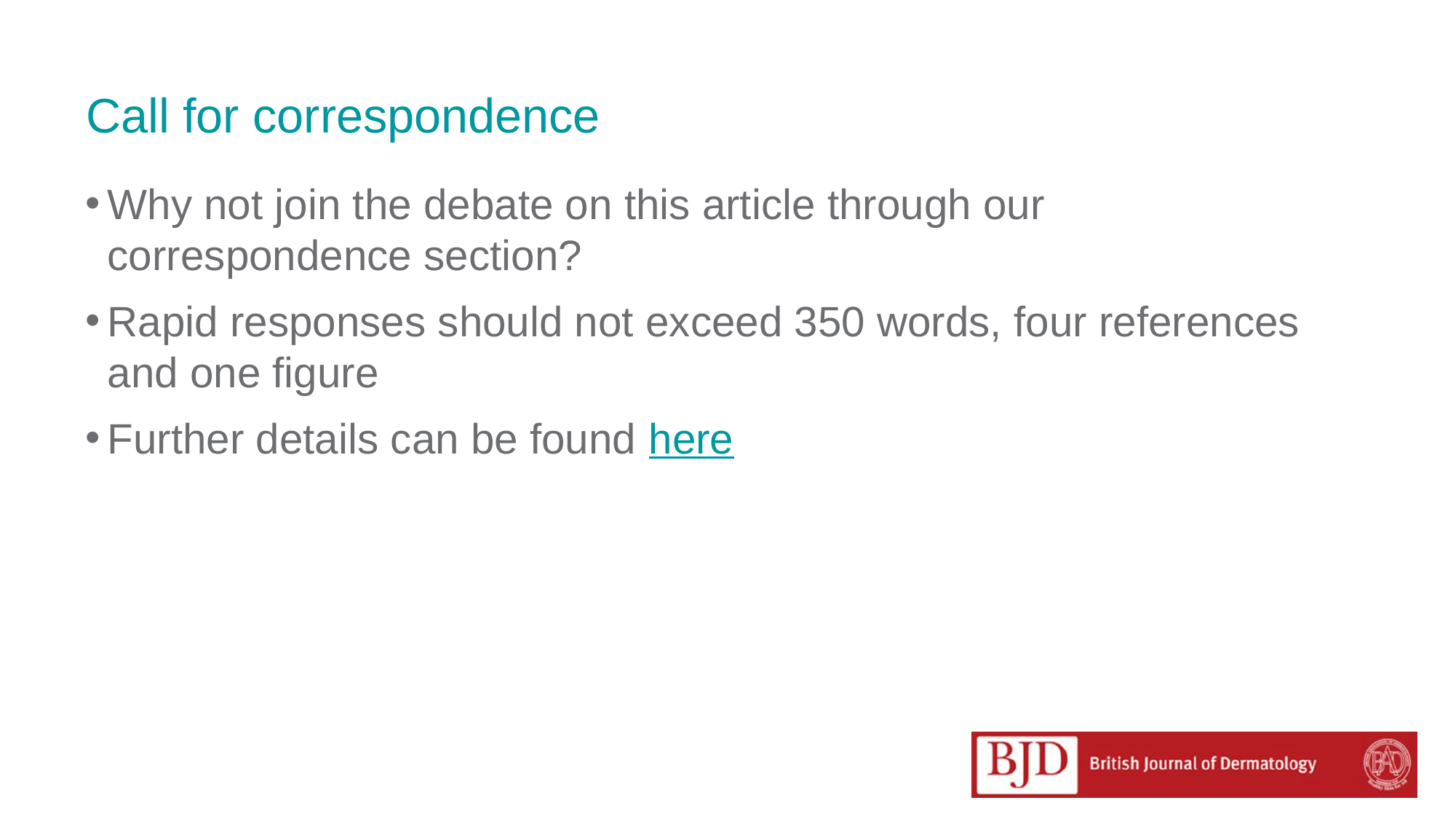

# Call for correspondence
Why not join the debate on this article through our correspondence section?
Rapid responses should not exceed 350 words, four references and one figure
Further details can be found here
